# Cigarette Smoking and Lung Cancer: Pediatric Roots

**DOI:** 10.1155/2012/790841

**Published:** 2012-08-30

**Authors:** Norman Hymowitz

**Affiliations:** Department of Psychiatry, UMDNJ-New Jersey Medical School, 183 South Orange Avenue, Newark, NJ 07103, USA

## Abstract

A vast array of data suggests that early age of smoking onset enhances the risk for development of lung cancer in adulthood. Initiation of smoking at a young age may influence the development of lung cancer because of its effect on duration of smoking. Early onset of smoking also may serve as an *independent* risk factor. It may increase the likelihood that smoking occurs during a critical period of development that enhances susceptibility to the adverse effects of cancer causing agents in cigarette smoke, thereby facilitating the initiation of the carcinogenic process. While evidence for the latter hypothesis derives from a variety of sources, definitive *proof* has proven elusive. Whether or not early age of smoking serves as an *independent* risk factor for lung carcinogenesis, the consensus of the public health community is that prevention of smoking onset at a young age and early cessation are keys to stemming the current lung cancer pandemic. Population approaches to tobacco prevention and control, such as measures contained in the World Health Organization Framework Convention Tobacco Control Treaty, offer the best opportunity, on the scale needed, to create a smoke-free world and bring an end to the pandemic of tobacco-related disease.

## 1. Introduction

Cigarette smoking has been called a *pediatric disease* [1]. Worldwide, between 82,000 and 99,000 young people begin smoking every day, 80% of them from low-income countries [2]. If current trends continue, more than 200 million young people under the age of 20 will die prematurely from tobacco-related diseases. More immediate effects on child health include higher rates of cough, fatigue and shortness of breath, and shortness of breath on exertion than youth who do not smoke. Those who smoke also are more prone to allergies, respiratory and ear infections, enhanced risk of asthma, and impaired lung growth [2].

In addition to immediate effects of cigarette smoking, early age at initiation of smoking increases the risk of lung cancer [3] and cardiovascular disease [4] during their life time. Of the organ sites at which smoking is known to cause cancer, smoking-associated genotoxic effects have been found for oral nasal, esophagus, pharynx, lung, pancreas, myeloid organs, bladder/ureter, and uterine cervix [2]. In addition to permanent changes in DNA, the reversibility of cancer risk after smoking cessation implies a role for epigenetic factors in carcinogenesis [2].

Initiation of cigarette smoking in childhood and adolescence plays a role in the development of lung cancer by virtue of its contribution to duration of smoking and life time exposure to smoking-related carcinogens. Early age at onset of smoking also may serve as an *independent* risk factor for lung cancer. As indicated below, there may be a critical period in which lung tissue is particularly susceptible to the first stage of carcinogenesis. The importance of understanding the role which early age of smoking plays in lung cancer development later in life resides in furthering our understanding of the carcinogenic process as a guide to curbing the current worldwide pandemic of lung cancer. To this end, the present paper takes a critical look at evidence that suggests that early age at smoking onset may serve as an *independent* risk factor for lung carcinoma.

Whether or not early age at the onset of smoking is an *independent* risk factor for lung cancer or exerts its effect solely by contributing to life-long exposure to cigarette smoke, there is an urgent need to protect young people from a lifetime of addiction and tobacco-related disease. By doing so, the public health and medical communities will have taken a bold step on the scale required to curb, if not end, the current worldwide lung cancer pandemic. To this end, population approaches to tobacco prevention and control are discussed.

### 1.1. Tobacco and the Lung Cancer Pandemic

*“The magnitude of excess lung-cancer risk among cigarette smokers is so great that the results can not be interpreted as arising from an indirect association of cigarette smoking with some other agent or characteristic, since the hypothetical agent would have to be at least as strongly associated with lung cancer as cigarette use; no such agent has been found or suggested.” [5]. *

The use of tobacco has been traced to early American civilizations, where it was first cultivated in 6000 BC and used in religious rites and ceremonies [6]. In 1492, Columbus and his crew observed natives lighting rolls of dried leaves, which they called *tobaccos *(cigars), and “swallowing” the smoke. Soon after, Juan Ponce de Leon brought tobacco to Portugal, where it was grown on Portuguese soil. In 1565, Sir Walter Raleigh introduced smoking to England and, ultimately, the growth of world trade led to the spread of tobacco to every corner of the globe [7]. By the mid-17th century, every major civilization had been introduced to tobacco smoking, laying the foundation for the 20th-century pandemic of tobacco-related morbidity and mortality [8].

Ironically, the ancients, as well as well as physicians in the 16th, 17th, 18th, and 19th centuries, strongly believed in tobacco's medicinal value [6]. During the plague of London in 1665, tobacco chewing was considered the most effective prophylactic measure against infection. In Philadelphia, where 10% of the population died of yellow fever in 1793, men, women, and children smoked cigars and drank beer as protection against the “American plague.” [6].

Scientific evidence against tobacco began to emerge early on. In 1670, the Dutch anatomist, Keckering, described the results of autopsies of heavy smokers. Perhaps, his observation that the “lungs were dried-out and almost friable” was among the first observations of the association between smoking and diseases of the lung. An examination of the issue of tobacco smoking by the Medical School of Paris in 1689 concluded that tobacco smoking shortens life. In 1761, the English physician, Jon Hill, reported an association between smoking and cancer of the nose, and in 1795, an article appeared in a medical journal linking pipe smoking with cancer of the lip [6].

It was not until the 20th century that the link between smoking and cancer was recognized and firmly established. In 1920, Broders published an article on the association between tobacco use and cancer of the lip. In 1928, Lombard and Doering reported that smoking was more common among cancer *cases* than nonsmoking *controls*. A 1940 case-control study by Muller in Germany suggested a link between smoking and lung cancer, but the message was largely lost as the medical community was distracted by World War II [6].

Prior to the 20th century, the occurrence of lung cancer was a rare event, and the early studies failed to command the attention of the medical community [8, 9]. However, in 1959, the Surgeon General of the United States concluded that “the weight of the evidence at present implicates smoking as the principle etiological factor in the increased incidence of lung cancer [10].” This conclusion was based on (1) the observation that the worldwide incidence of lung cancer was increasing dramatically, [8, 9], (2) seminal case-control studies by Doll and Hill [11] and Wynder and Graham [12], and (3) subsequent prospective studies by Doll and Hill [13] and Hammond and Horn [14]. Landmark reports by the Royal College of Physicians in 1962 [15] and the Advisory Commission to the Surgeon General of the United States in 1964 [16] provided indisputable proof that cigarette smoking lay at the heart of the worldwide increase in deaths from lung cancer.

As noted by Proctor, [9] lung cancer kills about 1.5 million people per year globally, and the total is expected to increase to nearly 2 million per year by 2020 or 2030. About 95% of those deaths are preventable, since lung cancer is primarily caused by the inhalation of smoke from cigarettes, second-hand smoke as well as mainstream smoke. Despite this, Proctor suggests that the enormous toll that tobacco use and smoke exposure exact on people throughout the world is likely to continue to rise throughout the 21st century [9]. His prediction is based on several factors: (1) the ability of cigarette making machines to “crank out” 20,000 cigarettes per minute; (2) the billions of dollars spent per year on marketing and advertising; (3) the growing popularity of cigarettes smoking among women, as well as men, in countries around the globe; (4) the addictive nature of nicotine. The harm done to date, as well the possibility of greater harm in the future, gave credence to the observation that “the cigarette is the deadliest artifact in the history of human civilization.” [9].

### 1.2. Age of Smoking Onset

*“Unfortunately, early use of tobacco has substantial health risks that begin almost immediately in adolescence and young adulthood, including impairment to the respiratory and cardiovascular systems. Many of the long-term diseases associated with smoking, such as lung cancer, are more likely among those who begin to smoke earlier in life.” [17]. *

The vast majority of adult smokers in the United States (more than 80%) [17] and elsewhere around the globe [7] report that they began smoking in their youth. If young people do not begin smoking by their late teens, they are unlikely to smoke as adults [17]. In the United States, the peak years for smoking onset are in the 6th and 7th grades, or between the ages of 11 and 13 [17]. A considerable number start even earlier [18]. In a nation-wide *Monitoring the Future *survey, 8.8% of 8th grade students reported having first smoked by the 5th grade (age 10 and 11 years old) [19]. Of those who experiment with smoking, more than one-third become daily smokers before they finish high school [19]. 

The results from the 2010 National Survey on Drug Use and Health in the United States indicate that each day approximately 3,800 young people less than 18 years of age smoke their first cigarette, and about 1,000 youth in that age group become daily smokers [20]. Moreover, youth become addicted to cigarettes far sooner than previously believed, [21] with some youths revealing signs of dependence within a day of first inhaling. This may explain why three out of four regular smokers in high school have already tried to quit but failed [21].

In contrast to past years when young boys in the United States were more likely to smoke than girls, now an equal number of girls and boys begin smoking every day. About 20% of each gender smoke in high school [22]. In many other countries, such as those in Asia, Africa, and the Middle East, smoking still is much more prevalent among males than females, [7] as is the incidence of lung cancer later in life [7]. However, the number of girls who smoke is increasing throughout the world [21]. In many countries, the number of female adolescents who smoke now outnumbers the number of boys who smoke [22]. This will no doubt contribute to a major increase in the global incidence of lung cancer among females in the 21st century.

Large scale epidemiological studies have shown that early age of smoking onset heightens the risk of developing lung cancer later in life, (e.g., [23–29]). A 1990 case-control study by Peto et al. [29] for example, showed that male and female smokers who started smoking before the age of 15 years had double the risk of lung cancer of those who started at age 20 years or more. It is of interest to determine whether early age of smoking onset affects the relative risk of developing lung cancer solely by contributing to lifetime exposure to tobacco smoke [24, 25] or also by exerting an *independent* effect that enhances the relative risk of lung cancer in adulthood [23].

Hegmann and colleagues [30] were among the first to suggest that early age at smoking initiation may have an effect on lung cancer risk over and above its contribution to intensity and duration of tobacco smoke exposure. The investigators used data from a population-based case-control study with 283 histologically confirmed lung cancer cases matched to 3,282 random controls to determine whether age at initiation of smoking has an *independent* influence on the occurrence of lung cancer. After controlling for age, sex, and amount of tobacco exposure, men who began to smoke before age 20 had a higher risk of developing lung cancer than men who began smoking at age 20 or older [30]. For women, the increase in risk continued until age 25 compared to women who began smoking at 26 years of age or older. An increased risk of lung cancer also was found among women who initiated smoking at ages 19–25 compared to those who began to smoke after 25 years of age [30].

Khuder et al. [31] used case-control methodology to examine the effect of cigarette smoking intensity, age at initiation, duration of smoking, and quitting on the development of different histological types of lung cancer in men. Logistic regression analyses showed that early age of smoking initiation (<16 years) significantly increased the risk of small cell carcinoma. Quitting smoking reduced the risk of squamous cell and adenocarcinoma, but did not affect the risk of small cell lung cancer [31].

The investigators concluded that an early age of smoking is associated with an overall increased risk of lung cancer [31]. The highest relative risk was observed for small cell carcinoma among those who started before age 16 compared to those who started smoking after 20 years of age. The investigators speculated that this association might be related to a specialized cell population in the bronchus of young individuals and other features of the developing lung of the child (e.g., susceptibility to airway closure and high peripheral resistance). Since the findings were based on multivariate analyses in which duration and intensity of smoking were controlled, the authors concluded that their study, like the above mentioned study by Hegmann et al. [30], established an i*ndependent* effect of early age at smoking onset for carcinoma of the lung [31].

Wiencke and associates [32] shed further light on the putative i*ndependent* effects of age at onset on the risk of lung cancer. They sought to determine whether DNA adducts formed as a consequence of smoking can be used as a *dosimeter *for cancer risk. They measured the adduct levels in nontumorous lung tissue (*n* = 143 patients) and blood mononuclear cells (*n* = 54) from patients with lung cancer. They also collected data from patients on their history of smoking. For current smokers, they collected information about age of smoking initiation, number of cigarettes smoked per day, number of years smoked, and number of pack years (number of cigarettes × duration of smoking/20). For former smokers, they also obtained information about years since quitting. Negative binomial regression models were used to assess the importance of smoking exposure variables on DNA adducts in lung tissue and blood mononuclear cells. Separate models were used for analyzing DNA adducts in current and former smokers. In current smokers, the number of cigarettes smoked per day was the most important variable. In exsmokers, the age at smoking initiation was most significant. Data derived from nontumerous lung tissue and blood mononuclear cells were highly correlated [32].

To further explore the role of cigarette smoke exposure on DNA adduct levels in lung tissue, they constructed regression models that simultaneously tested the effects of several smoking variables on adduct levels. Several variables differed significantly in current versus exsmokers. The intensity of smoking (cigarettes smoked per day) was positively associated with adduct levels in the lungs of current smokers but was not statistically associated with adduct levels in the lungs of exsmokers. In former smokers, a statistically significant negative correlation between adduct levels and the age at smoking initiation was found, but the variable was not important for current smokers.

The investigators proposed two general explanations for the results in former smokers: (1) decreased adduct removal through DNA repair and cell turnover or (2) increased adduct accumulation. Under the first scenario, early-age smoking initiation during a time of rapid lung growth and development may induce long-lasting physiologic changes that impair the removal of damaged bases in the DNA. In current smokers, the higher adduct levels and influence of recent smoking may mask the effects of early-age smoking and impaired adduct removal [32].

Alternatively, very young smokers may be markedly susceptible to adduct formation but have normal rates of adduct removal. Hence, young smokers may accumulate more damaged DNA that, even with normal repair, is demonstrable many years after smoking cessation. This stands in contrast to smokers who begin to smoke later in life [32]. The authors noted that their findings and hypotheses were consistent with earlier animal studies of the molecular biology of lung cancer [33].

Hirao and colleagues studied the relationship between early smoking initiation and somatic mutation [34]. They noted that the short arm of chromosome 3 may harbor a novel oncogenic (tumor suppressor) locus that is important in the genesis of lung cancer. The region at 3p21 is believed to contain a locus that is sensitive to loss of heterozygosity (i.e., the chromosome loses some repair capabilities) from the actions of tobacco smoke carcinogens. After examination of lung tissue from 219 lung cancer cases, they concluded that loss of heterozygosity in 3p21 may be an early molecular event in non-small cell lung cancer. Loss of heterozygosity induced in developing lung tissue may result in propagation of clonal regions, yielding large fields of cells with loss of heterozygosity [33]. Later in life, a second somatic “hit” (mutation) on the other chromosome by prolonged smoking then leads to homozygous loss of this important tumor suppressive gene and the aggressive development of cancer cells. 

Based on the above findings, Wiencke and Kelsey [35] hypothesized that teen smoking may serve as a *critical period* for *field cancerization* and lung cancer susceptibility. Smoking-related genetic alterations of the respiratory epithelium occurring early in life may confer an increased risk of lung cancer independent of smoking intensity and duration of smoking. They proposed that normal developmental processes related to lung growth following puberty, in concert with exposure to tobacco smoke, may promote abnormal clonal proliferation, initiating a process termed *field cancerization*. The abnormal clonal fields caused by smoking may form the basis of lethal cancers later in life [35].

Multistage models of lung cancer development highlight the importance of early smoking-induced mutations and provide additional support for an independent effect of early age at smoking onset. The “multistage model” hypothesis for carcinoma induction suggests that a few changes, each heritable when somatic cells divide, are needed to alter a normal epithelial cell into a cancer progenitor cell [23]. During this early phase, transformational events occurring cell by cell may be separated from each other by several years. The biologic nature of the early stages of carcinogenesis may play a decisive role in the development of lung cancer by initiating a continuum of changes that occur during the later, more aggressive, stages of carcinogenesis [23]. 

Modeling of data for male and female participants in the European Prospective Investigation into Cancer and Nutrition [EPIC] study [36] produced results which are supportive of the critical period hypothesis and an *independent* contribution to lung cancer risk of early age at smoking onset [36]. The model contained the rate-limiting stages of carcinogenesis (initiation, promotion (clonal expansion of initiated cells), malignant transformation, and a lag time for tumor formation). To investigate the relative importance of smoking rate, age at start of smoking, and smoking duration on lung cancer risk, a series of simulations were performed using the best estimates of the joint fit of EPIC males and females. The findings indicated that with respect to lung cancer risk, age of smoking initiation and duration of smoking mattered most. Smoking intensity made a smaller difference [36].

The investigative team also modeled the influence that a smoking-dependent first mutation rate would have on the risk patterns. They found that the ratio of the excess relative hazards was largest for those model smokers who started smoking earlier. The ratio was highest for smokers who started at young ages and who only smoked for short periods of time. That is, for individuals who started smoking 20 cigarettes per day at age 15 years and who smoked for five years, the ratio in excess relative hazard was about 3.5. This heightened risk for lung cancer, given the short duration of exposure, is consistent with the influence of early age smoking, perhaps at a critical period of development, on initiation and clonal expansion that may set in motion the process of life threatening malignancy in adulthood [36]. 

### 1.3. Prevention of Lung Cancer: A Global Perspective

*“Preventing smoking and smokeless tobacco use among young people are critical to ending the tobacco epidemic. Since the first Surgeon General's report on youth in 1994, the basis for concern about smoking during adolescence and young adulthood has expanded beyond the immediate health consequences for the young smoker to a deeper understanding of the implications for health across the life span from early tobacco use.” [37]. *

Whether or not age of smoking onset is an independent risk factor for lung cancer rates later in life or influences lung cancer risk primarily by contributing to duration of exposure to tobacco smoke, there is overwhelming consensus that the key to preventing lung cancer and stemming the 21st century pandemic lies in preventing smoking onset in young people and helping those who start to quit. The most wide-reaching initiative for stemming tobacco use and smoke exposure in young people on the scale needed to impact worldwide rates of lung cancer and other chronic diseases is the World Health Organization Framework Convention on Tobacco Control (WHO FCTC) [38]. The WHO FCTC is a legally binding global treaty that provides the foundation for countries to implement and manage tobacco control programs to address the growing epidemic of tobacco use. As of May, 2011, the WHO FCTC had 173 parties covering 87% of the world population [39]. 

As noted by Morello in her recent review of policies to prevent tobacco use and exposure in children, [40] the WHO FCTC represents a paradigm shift in developing a regulatory strategy to address addictive substances. It asserts the importance of demand reduction strategies as well as supply issues. The FCTC provides seven primary strategies for worldwide tobacco control: (1) increase tobacco taxes; (2) protect citizens from tobacco smoke exposure in work places, public transport, and indoor public places; (3) mandate rotating health warnings on tobacco packaging that includes pictures or “pictograms” and ban the use of misleading terms such as “light” and “mild”; (4) increase public awareness and education about tobacco; (5) enact comprehensive bans on tobacco advertising, promotion, and sponsorship; (6) eliminate all forms of illicit trade of tobacco products; (7) prohibit the sale of tobacco products to minors, the sale of single cigarettes, and the distribution of free tobacco products.

To help countries fulfill their WHO FCTC obligations, the WHO introduced the MPOWER package of six evidence-based tobacco control measures that are proven to reduce tobacco use and save lives [41]. The WHO MPOWER Frame Work includes the following key initiatives: (1) Monitor tobacco use and prevention policies; (2) Protect people from tobacco smoke; (3) Offer to help to quit smoking; (4) Warn about the dangers of tobacco use; (5) Enforce comprehensive restrictions on tobacco advertising, promotion, and sponsorship; (6) Raise taxes on tobacco.

This global approach to tobacco control brings together a widerange of participants. They include world, national, and community leaders, governmental bodies, voluntary agencies, advocates, educators, and health care providers, particularly those that treat and care for young people. With respect to the latter, it is critical that child health care providers address tobacco prevention and control in parents and care takers, as well as in children, adolescents, and young adults. Parents have a major impact on the smoking behavior of children, and when they quit or refrain from smoking, create a smoke-free household, and/or talk with young people about how they can resist pressure to smoke, they contribute to the *denormalization *of smoking and provide a model of a smoke-free lifestyle for their children [17].

The decline in smoking rates and lung cancer deaths in a number of developed countries [42] offers firm evidence for the efficacy of the WHO FCTC recommendations. While the United States is not a signatory of the Framework Convention Treaty, it has embraced the spirit of the convention. Since publication of the first Surgeon General' Report in 1964, the United States has implemented, or is in the process of implementing, virtually all of the FCTC recommendations. A recent comparative modeling study of the impact of reduced smoking on lung cancer mortality in the United States during 1975–2000 suggests that approximately 795,852 lung cancer deaths were averted due to changes in smoking behavior among American men and women [42]. Approximately 2,504,043 lung cancers could have been averted had tobacco control efforts been completely effective in eliminating smoking as of 1965 [42].

An editorial by Glynn noted that since 2000, the pace of tobacco control in the United States has accelerated [43]. The Affordable Care Act has made tobacco dependence treatment available to millions more smokers who want to quit. The Family Smoking Prevention and Tobacco Control Act has given the United States Food and Drug Administration authority to regulate tobacco products and to protect both youth and adults from their use [43]. With the planned addition of graphic warning labels on cigarette packs later in the year, an intervention which may discourage young people from taking up the habit, the United States has fully embraced, if not ratified, the provisions of the Framework Convention on Tobacco Control. There is a strong need to maintain the momentum. For the first time in decades, the American people, as well as those throughout the world, are able to get a glimpse of a “light at the end of the tunnel.” 

## 2. Discussion

The studies described above support the hypotheses that early age at smoking onset serves as an *independent *risk factor for lung cancer and there may be a *critical period* when lung tissue is particularly susceptible to smoking-induced carcinogenesis. However, definitive *proof *remains elusive, and there is need for additional research in areas such as the molecular biology and epidemiology of lung cancer. It should be noted that a number of cohort studies (e.g., [44–46]) failed to support the above mentioned hypotheses. In an analysis of data from the American Cancer Society Prevention Study I that entailed 12-year followup of one million men and women, Burns et al. [45] stratified data based on different ages of smoking initiation while holding constant duration of smoking. They failed to find an independent effect of age at initiation. Burns et al. concluded that “the major contribution of early age initiation to increased risk of lung cancer is mediated through the longer duration of smoking at any given age that occurs with earlier age of initiation. Whatever *independent *effect that early age of smoking onset may have on risk of lung cancer later in life was considered modest.” [45].

Like so many other issues in *life course epidemiology *[47], our understanding of the effects of events that occur early in life on a disease which does not occur until many years later has proven to be quite elusive. A number of factors may contribute to the difficulty of determining whether or not age at smoking initiation is an *independen*t risk factor for lung cancer later in life. As noted by Weincke et al., [32] the cumulative effects of a lifetime of smoking, particularly for those who never quit, may mask factors that played a significant role early on. Moreover, people who start smoking at an early age tend to differ in important ways from those who start smoking later, presenting additional barriers to parsing out the effects of early age smoking *per se* on lung cancer. Early onset smokers tend to smoke more cigarettes per day, inhale deeper, and are less likely to quit smoking than those who start smoking later [48, 49]. They typically are of lower socioeconomic status, have more difficulty in school, and are more likely to have parents and siblings who smoke than nonsmokers or those who initiate smoking at a later age [50]. Hence, young people who start smoking at an early age not only smoke for a longer duration than those who start smoking later, they also smoke at a higher intensity and may be exposed to second-hand smoke earlier in life [51, 52]. By virtue of their lower socioeconomic status, they may ultimately work in occupations in which their exposure to tobacco smoke and other sources of carcinogens is greater than those who started smoking at a later age [53].

Genetic factors also may play a role. Specific chromosome loci associated with enhanced susceptibility to nicotine addiction and lung cancer have been identified [54]. Despite the difficulty in definitively parsing the influence of early factors on lung cancer development later in life, the thrust of the data collected to date underscores the importance of reaching out to vulnerable youth, preventing smoking onset at an early age, and helping youth who smoke to quit as soon as possible. Many developing countries are now only in the beginning stages of the tobacco epidemic, [55] and they are in a position to protect their youth from lung cancer and other tobacco-related diseases. As recommended by the WHO FCTC, they must enact comprehensive tobacco control plans, initiate community and nation-wide anti-tobacco campaigns, inform their public about the health threat cigarettes and other forms of tobacco pose, and take actions to protect their citizenry from second-hand tobacco smoke exposure. By doing so, they may avert the dramatic rise in lung cancer deaths and other forms of tobacco-related mortality and morbidity with which the United States and other developed countries still are coping. 
